# The regulatory effects of PD-1/PD-L1 inhibitors on bone metabolism: opportunities and challenges in osteoporosis management

**DOI:** 10.3389/fimmu.2025.1630751

**Published:** 2025-07-25

**Authors:** Jia-Wen Wang, Mu-Wei Dai, Jia-Hui Liu

**Affiliations:** Department of Orthopedics, The Fourth Hospital of Hebei Medical University, 12 Health Road, Shijiazhuang, Hebei, China

**Keywords:** PD-1/PD-L1 inhibitors, bone metabolism, immune-related adverse events, osteoclasts, osteoblasts, RANKL, Wnt signaling, bone biomarkers

## Abstract

Programmed death-1 (PD-1) and its ligand PD-L1 inhibitors have become pivotal agents in cancer immunotherapy, demonstrating significant efficacy across multiple malignancies. However, beyond regulating T cell activation, the PD-1/PD-L1 axis also exerts complex and critical effects on bone metabolism. Notably, both clinical observations and mechanistic studies have revealed a paradox: on one hand, PD-1/PD-L1 blockade appears to confer bone-protective benefits; on the other hand, it has been associated with bone-related adverse events (AEs) in up to 69% of patients, including pathological fractures and vertebral compression fractures. This review comprehensively explores the bidirectional regulatory effects of the PD-1/PD-L1 pathway on bone metabolism and investigates the underlying mechanisms contributing to these contradictory findings. The discrepancies may be attributed to a combination of clinical variables, microenvironmental conditions, cell-specific responses, and intricate interactions among multiple signaling pathways, including the Wnt/β-Catenin pathway and the PD-L1–PKM2 axis. We further examine the pathophysiological basis of osteoporosis and fragility fractures occurring during PD-1/PD-L1 inhibitor therapy, and argue for their recognition as a subclass of immune-related adverse events (irAEs). Finally, we propose a framework for bone health surveillance and stratified prevention strategies aimed at preserving antitumor efficacy while improving skeletal health and quality of life—offering novel insights into osteoporosis prevention and management in the context of immune checkpoint inhibition.

## 1 Introduction

Inhibitors targeting programmed death-1 (PD-1) and its ligand PD-L1 have profoundly reshaped cancer therapy. Since the first PD-1 inhibitor received regulatory approval in 2014, these agents have significantly improved overall survival (OS) and progression-free survival (PFS) rates (1), becoming standard treatments for a wide range of malignancies, including non-small cell lung cancer (NSCLC), melanoma, head and neck squamous cell carcinoma, and renal cell carcinoma (2).

Beyond their immunomodulatory effects on T cells, the PD-1/PD-L1 axis plays a multifaceted role in bone metabolism. Murine models with PD-1 or PD-L1 gene knockout exhibit pronounced osteoporotic phenotypes, including decreased trabecular bone volume, disrupted microarchitecture, elevated osteoclastogenesis, and increased RANKL/OPG ratios (3). These outcomes are mediated via multiple signaling cascades that finely regulate bone-resorbing and bone-forming cells (4–6). However, the complexity of these mechanisms has led to conflicting results. While some clinical studies suggest that PD-1/PD-L1 inhibitors exert bone-protective effects (4), others report the opposite, documenting bone-related adverse events in patients receiving immune checkpoint inhibitors (ICIs) (7, 8), even as some individuals maintain stable bone mineral density (9).

At the molecular level, similarly inconsistent findings are observed. Some investigations describe a pro-osteogenic role for PD-1/PD-L1 blockade (10), while others note inhibitory effects on osteoblast differentiation (6). These contradictions are likely influenced by a convergence of factors, including patient-specific clinical features, the immune microenvironment, cell-type-specific responses, soluble PD-1/PD-L2 activity (11), and the bidirectional nature of the Wnt/β-Catenin pathway (5, 12) and PD-L1–PKM2 metabolic signaling (13).

Furthermore, clinical evidence links PD-1/PD-L1 inhibitors to a heightened risk of pathological fractures, vertebral compression fractures, and femoral neck fractures (14), contributing to a cumulative incidence of bone-related adverse events as high as 69% (15). Despite their prevalence and clinical significance, conditions such as osteoporosis and fragility fractures are not formally recognized as immune-related adverse events (irAEs) (16), underscoring a gap in clinical classification and management.

**Figure 1:**
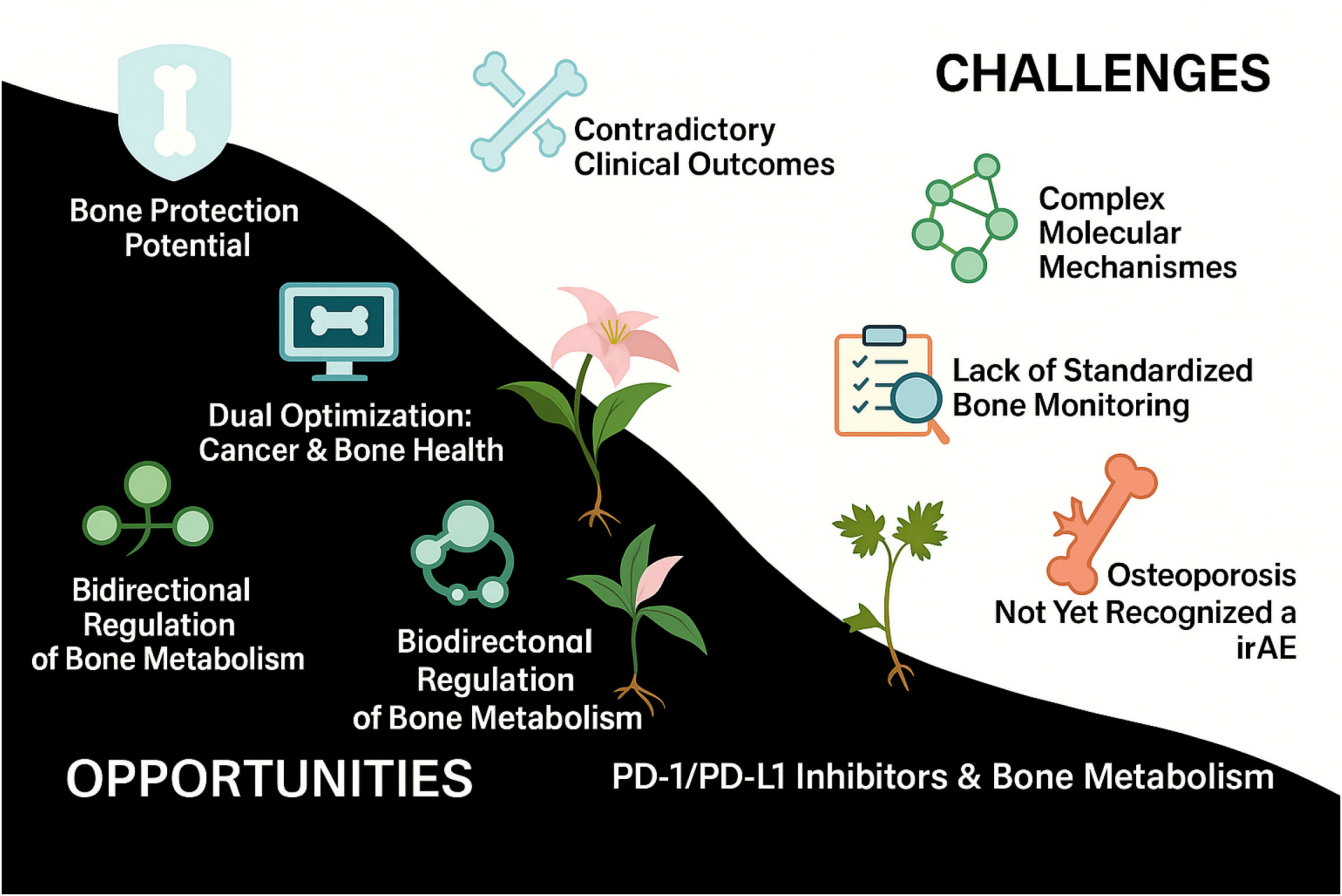
Opportunities and challenges of PD-1/PD-L1 inhibitors in bone metabolism. This schematic illustrates the dual impact of PD-1/PD-L1 inhibitors on bone metabolism. The left panel highlights the potential benefits, including bone-protective effects, bidirectional immuno-skeletal regulation, and dual optimization of tumor control and bone health. In contrast, the right panel outlines key challenges, such as inconsistent clinical outcomes, complex molecular mechanisms, the absence of standardized bone surveillance protocols, and the underrecognition of osteoporosis as an immune-related adverse event (irAE).

**Figure 1** outlines the opportunities and challenges associated with PD-1/PD-L1 blockade in bone metabolism, emphasizing its potential protective effects alongside its regulatory complexity and the under-recognition of skeletal irAEs such as osteoporosis.

To elucidate the dual role of PD-1/PD-L1 inhibitors in bone metabolism and explore their implications in osteoporosis prevention, this review addresses the following key questions:

1. What accounts for the contradictory findings regarding the effects of PD-1/PD-L1 inhibitors on bone metabolism? Why do some studies report bone-protective outcomes (e.g., reduced resorption markers and preserved BMD), while others observe increased bone-related AEs such as vertebral fractures? What clinical factors (e.g., baseline patient characteristics, cancer type, treatment regimens) may underlie these discrepancies?
2. How does the PD-1/PD-L1 pathway achieve bidirectional regulation of bone metabolism? Through which signaling networks does it affect osteoclast differentiation/function and osteoblast activity/mineralization? What roles do the Wnt/β-Catenin pathway and the PD-L1–PKM2 axis play in this context, and how do these mechanisms reconcile clinical inconsistencies?
3. How can the dual regulatory features of PD-1/PD-L1 blockade be translated into osteoporosis prevention strategies? What are the evidence-based approaches for integrating bone health monitoring and stratified prevention during ICI therapy? What justifies the inclusion of osteoporosis and fragility fractures as irAEs, and how can this reclassification enhance both cancer and skeletal outcomes?

## 2 Effects of PD-1/PD-L1 inhibitors on bone metabolism

PD-1/PD-L1 immune checkpoint inhibitors have become a cornerstone of modern cancer immunotherapy. However, their effects on bone metabolism remain underrecognized and exhibit contradictory findings across studies. Gassner et al. reported that in cancer patients without bone metastases, treatment with PD-1/PD-L1 inhibitors led to a significant early reduction in the bone resorption marker CTX (from a baseline mean of 0.51 ng/ml to 0.42 ng/ml at week 3), while bone formation markers such as PINP and osteocalcin (OCN) increased after 4 months of treatment, suggesting a bone-protective effect (4). Conversely, Pantano et al. observed a marked increase in CTX-I levels and a downward trend in PINP levels after 3 months of immune checkpoint inhibitor (ICI) therapy, which correlated with poor treatment response and decreased survival (7).

Moreover, some studies have documented adverse skeletal events during ICI treatment, including vertebral compression fractures and osteolytic lesions (8). In contrast, others have reported relatively stable bone mineral density (BMD) in ICI-treated patients compared to non-ICI counterparts, suggesting a long-term bone-preserving effect (9). These conflicting outcomes underscore the complex and multifactorial nature of PD-1/PD-L1 blockade on bone metabolism, likely influenced by clinical heterogeneity such as age, sex, tumor type, treatment regimen (monotherapy vs. combination therapy), and baseline skeletal health (17).

Importantly, there remains a lack of standardized bone health monitoring protocols for patients receiving ICIs, potentially delaying the detection and intervention of subclinical bone metabolic disorders (18). This gap may contribute to skeletal-related adverse events (SREs), ultimately impairing therapeutic efficacy and quality of life. Therefore, the impact of PD-1/PD-L1 inhibition on bone health warrants greater clinical attention and systematic evaluation.

## 3 Regulatory effects of PD-1/PD-L1 pathway and inhibitors on osteoclasts

While the PD-1/PD-L1 axis plays a pivotal role in immune regulation, it also significantly influences bone metabolism, particularly osteoclast differentiation and function. Genetic deletion of PD-1 or PD-L1 leads to osteoporotic phenotypes, implicating this pathway in the maintenance of bone homeostasis (3). Within the tumor microenvironment (TME), upregulation of PD-L1 and CCL2 facilitates osteoclastogenesis by activating the JNK pathway and enhancing CCL2-mediated RANKL signaling, thereby promoting bone resorption (5). Additionally, soluble PD-1 (sPD-1), which is elevated in inflammatory settings, stimulates IL-17A production—a key mediator of osteoclast activation—resulting in accelerated bone destruction. In contrast, PD-L2 expression under inflammatory conditions appears to suppress osteoclastogenesis and confer bone-protective effects (11).

Osteoclasts, particularly in their activated state, can upregulate PD-L1 expression to inhibit T cell proliferation and cytotoxicity, contributing to an immunosuppressive microenvironment. This PD-L1 upregulation is itself modulated by pro-inflammatory cytokines such as IFN-γ and IL-6, forming a feedback regulatory loop (19, 20).

PD-1/PD-L1 inhibitors interrupt this axis and exert both direct and indirect effects on osteoclast activity. By inhibiting the JNK pathway, these agents reduce osteoclast proliferation and resorptive capacity, leading to decreased CTX levels (4, 5). They can also interfere with the STAT3/NFATc1 signaling cascade, impeding pre-osteoclast maturation and reversing osteoclast-mediated immunosuppression (20). This dual mechanism results in a bidirectional modulation of bone remodeling (4), with short-term treatment reducing TRAP+ osteoclasts by approximately 60%, lowering bone destruction scores by 40%, and decreasing CTX by 23%, while increasing PINP levels by 8% at 4 months (p = 0.02) (4, 5). For a detailed summary of these findings across preclinical and clinical contexts, see **Table 1**.

**Table 1:** Effects of the PD-L1/PD-1 Axis on Osteoclasts and Bone Metabolism.

Year	Model / System	Intervention & Main Indicators	Osteoclast / Bone Metabolism Findings	Mechanistic Insights	Ref.
2020	Lewis lung carcinoma bone metastasis mouse model; RAW264.7 cells	Anti-PD-1 (nivolumab 10 mg/kg) or Pdcd1–/–; TRAP staining, μCT, serum CTX-I/PINP	TRAP^+^ osteoclasts ↓ ≈60%; serum CTX-I ↓ (Day 8); bone destruction score ↓ 40%; pain alleviation	Tumor-derived PD-L1 activates PD-1 on osteoclast precursors → JNK → CCL2 ↑ → promotes RANKL-driven osteoclastogenesis; PD-1 blockade reverses this process	(5)
2022	PD-1–/– and PD-L1–/– mice; human/mouse BM cells	Gene knockout or neutralizing antibodies; RANKL/M-CSF induction	PD-1 KO: RANKL ↑, RANKL/OPG ratio ↑; TRAP^+^ osteoclasts ↑ 3–4-fold; BMD ↓	PD-1 pathway suppresses RANKL and osteoclast activation in inflammatory settings; its loss leads to excessive bone resorption	(3)
2023	Cohort of 2,532 cancer patients receiving ICIs	Observational cohort; endpoint: major fracture incidence	Fracture incidence 7.3 vs 3.6/100 person-years; adjusted HR ≈ 1.6 (95% CI: 1.2–2.2)	Enhanced T cell activation + corticosteroids may accelerate bone loss; highlights need for skeletal monitoring	(21)
2024	9 solid tumor patients on ICI monotherapy (no bone metastases) + ex vivo 3D bone model	PD-1/PD-L1 inhibitors (pembrolizumab, atezolizumab); dynamic bone turnover markers (CTX, PINP, OCN); PBMC–osteoblast/osteoclast coculture	CTX ↓ 23% at 1 month (p = 0.01); PINP ↑ 8% at 4 months (p = 0.02); ex vivo TRAP^+^ osteoclasts ↓ 50–70% dose-dependently	ICIs inhibit mature osteoclast differentiation, disrupting OC/OB coupling; net effect favors osteogenesis	(4)

However, prolonged ICI therapy has been associated with increased fracture risk, with adjusted hazard ratios nearing 1.6 (21), suggesting a shift toward net bone loss over time. Chronic inflammation during extended PD-1 blockade can enhance RANKL expression and osteoclast activation, further contributing to skeletal damage (3).

Wnt/β-Catenin signaling also plays a complex, bidirectional role in mediating the skeletal effects of PD-1/PD-L1 inhibition (**Table 2**). The canonical Wnt/β-Catenin pathway synergizes with short-term PD-1 blockade to suppress osteoclastogenesis and increase bone mass (5). In contrast, the non-canonical Wnt5a–Ror2 axis promotes osteoclast formation and reduces BMD, with Wnt5a overexpression in the TME antagonizing the bone-preserving effects of PD-1 inhibitors (12). Moreover, chronic PD-1 deficiency can suppress canonical Wnt signaling through sustained inflammation, leading to a 72% increase in osteoclast numbers and a 12% decrease in bone density (22).

**Table 2:** Bidirectional modulation by the Wnt/β-catenin pathway in ICI-treated patients: effects on osteoclasts and bone metabolism.

Year	Model / Population	Wnt/β-Catenin Regulation (Canonical vs Non-Canonical) + ICI	Osteoclast / Bone Findings	Mechanistic Insights	Ref.
2012	Wnt5a–/–, Ror2–/–, and WT mice	Non-canonical: Wnt5a–Ror2 axis	TRAP^+^ osteoclasts ↓ 45–60%; BMD ↑ 20–33%	Osteoblast-derived Wnt5a → Ror2^+^ osteoclast precursors → NFATc1 upregulation; tumor-bone microenvironmental Wnt5a may negate PD-1 blockade bone-protective effects	(12)
2020	Lewis lung carcinoma femoral metastasis model; nivolumab i.v.	ICI monotherapy: anti-PD-1	TRAP^+^ osteoclasts ↓ ≈60%; μCT BV/TV ↑ 29%; CTX-I ↓; pain relief	PD-1–SHP2 blockade → suppresses JNK–CCL2 cascade → inhibits osteoclast precursor differentiation; canonical Wnt activation may synergize	(5)
2021	Lung cancer (CMT167/MC38) + bone marrow fibroblasts (BMF)	Canonical inhibition: Tankyrase inhibitor XAV-939 + anti-PD-L1	TRAP activity ↓ ≈45% in co-culture; CD8^+^IFN-γ^+^ T cells ↑ 4.2-fold in combo group	BMF-induced β-catenin → PD-L1 ↑; XAV-939 inhibits β-catenin → PD-L1 ↓ → enhances anti-PD-L1 efficacy and suppresses osteoclasts	(40)
2022	Gastric cancer xenograft + dual antibody therapy	Canonical antagonism: High DKK1 (β-catenin inhibitor) + anti-PD-1	DKK1 overexpression → OC area ↑ 2.1-fold, OPG ↓; DKK1-mAb + anti-PD-1 → OC area ↓ 48%, bone metastases ↓ 40%	DKK1 suppresses β-catenin → M2-like TAM ↑, RANKL ↑; DKK1 neutralization restores β-catenin–OPG axis, suppresses osteoclasts and enhances ICI efficacy	(23)
2025	Pdcd1–/– mice under physiological conditions	PD-1 deletion → low canonical Wnt activity	Sex-specific: Male mice TRAP^+^ osteoclasts ↑ 72%, BMD ↓ 12%; β-catenin target Axin2 ↓	Chronic PD-1 loss → inflammation chemotaxis → inhibits β-catenin–OPG signaling → bone resorption imbalance; suggests prolonged/high-dose ICI or inflammatory context may reverse bone protection	(22)

To mitigate this deleterious effect, concurrent administration of anti-DKK1 antibodies—targeting inhibitors of canonical Wnt signaling—has shown promise in reducing skeletal damage and restoring bone homeostasis in the context of long-term ICI therapy (23).

## 4 Regulation of osteoblasts by the PD-1/PD-L1 pathway

Although the PD-1/PD-L1 pathway has been shown to promote osteogenic gene expression and calcium deposition (10), it can also inhibit osteogenic differentiation (6). This inhibition may occur through suppression of the SHP2 signaling pathway, which relieves its inhibitory effect on NF-κB activation, thereby enhancing osteoblast differentiation and bone formation (24). These seemingly contradictory effects may be related to the PD-1/PD-L1–PKM2 axis (13) (see **Table 3**). The balance within this axis plays a critical role: PD-L1 promotes osteogenic differentiation by upregulating RUNX2 expression, increasing ALP activity by 42%, enhancing mineralization by 30%, reducing inflammation, and promoting oxidative phosphorylation metabolism (10). In contrast, activation of PKM2 leads to increased tetramer formation, inhibition of glycolysis, a 60% reduction in RUNX2 expression, and a 35% decrease in ALP activity, collectively impairing osteoblast differentiation (25). Notably, PKM2 inhibition can reduce osteoclast numbers by 41%, improve oxidative metabolism and mitochondrial function, and enhance bone volume/tissue volume (BV/TV) ratio by 25%, thus promoting osteogenesis (26).

**Table 3:** Impact of the PD-1/PD-L1–PKM2 axis on osteoblast function and bone metabolism.

Year	Model / Intervention	Osteogenic Outcomes (ALP / Mineralization, etc.)	Mechanistic Insights	Ref.
2020	MC3T3-E1 + PKM2 activator DASA-58	RUNX2 mRNA ↓ 60%; ALP ↓ 35%; impaired osteogenesis	PKM2 tetramer formation ↑ → glycolysis ↓ → suppressed osteoblast activity	(25)
2022	hADMSC–osteoblast coculture ± PD-L1	PD-L1 treatment → RUNX2 ↑; ALP activity ↑ 42%; mineralized nodules ↑ 30%	PD-1/PD-L1 alleviates IL-6/TNF-α inflammation; suggests metabolic shift toward OxPhos	(10)
2024	Clinical ICI-treated cohort (n = 428)	Vertebral BMD ↑ 5.6% at 12 months; ALP/mineralization unchanged	ICIs mainly inhibit osteoclasts; limited direct osteoblast impact observed clinically	(4)
2025	BMSCs: si-PKM2 or TEPP46	PKM2-KD: BV/TV ↑ 25%, OC.N/BS ↓ 41%; TEPP46 showed similar effects	PKM2 inhibition promotes oxidative metabolism and mitochondrial restoration	(26)

Additionally, the bidirectional modulation of the Wnt/β-Catenin pathway influences osteoblast function and bone metabolism in patients receiving immune checkpoint inhibitors (ICIs) (see **Table 4**). Suppression of Wnt signaling significantly reduces bone mass (BV/TV by approximately 60%) and bone strength (by 33%), suggesting the need for bone-protective interventions during ICI therapy (27). Conversely, DKK1 can relieve Wnt inhibition, increasing osteoblast density (2.4-fold), osteoblast surface coverage (by 65%), and bone mineral density (by 9%). When combined with anti-PD-1 therapy, it further enhances antitumor immunity (28, 29), as β-catenin not only promotes osteogenesis but also induces PD-L1 expression (3–4-fold increase), contributing to a “self-limiting” immunosuppressive feedback loop. PD-1/PD-L1 blockade can reverse this suppression (30). From an ICI-centered perspective, PD-1 receptor activation promotes osteogenic markers via the ERK/β-catenin signaling pathway (ALP ↑ 61%, OCN ↑ 54%, mineralization ↑ 83%), whereas PD-1 blockade may weaken this osteogenic effect (10). Therefore, in cancer patients receiving ICIs, bone metabolism assessment must take into account the net outcome of these bidirectional regulatory mechanisms.

**Table 4:** Bidirectional effects of the Wnt/β-catenin pathway on osteoblast function in ICI-treated conditions.

Year	Tumor Model / System	Wnt/β-Catenin Regulation + ICI Treatment	Osteoblast/Bone Metabolism Outcomes	Mechanistic Insights	Ref.
2018	C57BL/6 mice (♀, 10 w)	Porcupine inhibitor LGK974 (5 mg/kg/day, 4 weeks) ± anti-PD-1 combo (RxC004/NIVO, public data)	Trabecular BV/TV ↓ ≈60%; cortical thickness ↓ 22%; MAR/BFR ↓ 40–45%; 3-pt bending strength ↓ 33%	Global Wnt inhibition → β-catenin suppression → Runx2/OPG ↓, Sost ↑; osteoblast inhibition with secondary resorption; suggests need for bone protection in ICI combination strategies	(27)
2020	U251 and GL261 glioma + ICG-001/anti-PD-1	Wnt3a/EGF → β-catenin ↑ → PD-L1 ↑ 3–4×; β-catenin/AKT inhibition or anti-PD-1 reverses effects	β-catenin–OE BMSC coculture: ALP/OCN ↑ 1.7×; PD-L1 suppresses CD8^+^ T activation; ICG-001 + anti-PD-1 restores CD8 function and maintains Runx2/Col1a1 → osteogenesis preserved	Canonical β-catenin promotes osteogenesis but induces PD-L1 → “self-limiting” immunosuppression; PD-1 blockade releases immune brake without impairing bone formation	(30)
2007 + 2021	SCID-rab myeloma model + B16F0/4T1	DKK1-neutralizing mAb (BHQ880/DKN-01) ± anti-PD-1	Myeloma transplant BMD ↑ 9% (vs −6%); OCN^+^ osteoblast density ↑ 2.4×; bone surface osteoblast coverage ↑ 65%; DKN-01 + anti-PD-1 maintained bone mass and inhibited tumors	DKK1 blockade → LRP5/6 reactivation → β-catenin–TCF–Runx2–OPG restored; osteoblast proliferation and anti-resorptive effects; also reduces MDSCs and activates NK/T cells → enhanced PD-1 efficacy	(28, 29)
2022	hADMSC-derived osteoblasts ± BMS-202	Exogenous PD-L1 / exosomes → PD-1 activation; BMS-202 inhibition	ALP ↑ 61%, OCN ↑ 54%, Ca²^+^ crystal area ↑ 83%; BMS-202 normalized osteogenesis index to control	PD-1 on osteoblasts → p-ERK/β-catenin ↑ → COL1A1–RUNX2 signaling ↑; low-inflammation microenvironment; co-culture with osteoclasts maintains bone homeostasis	(10)

Nevertheless, clinical evidence suggests that the primary bone-related effects of ICIs stem from inhibition of osteoclast activity and indirect promotion of bone formation, rather than direct osteoblast activation (4). These findings offer important implications for future osteoporosis management strategies.

**Figure 2:**
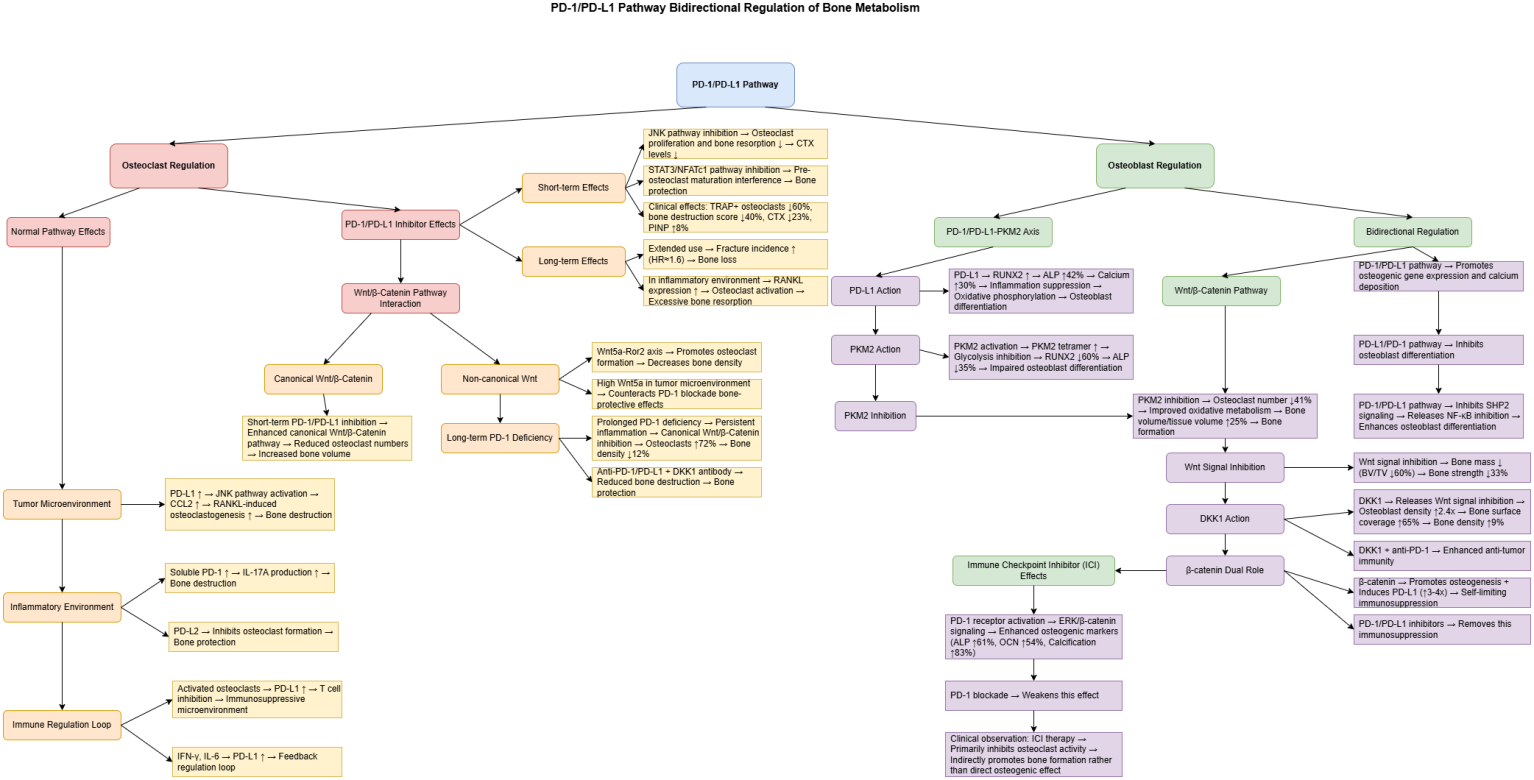
Bidirectional regulation of bone metabolism by the PD-1/PD-L1 pathway and the mechanisms of its inhibitors. This figure illustrates the regulatory network of the PD-1/PD-L1 pathway in bone metabolism. The left panel represents osteoclast regulation, while the right panel shows osteoblast regulation. Short-term administration of PD-1/PD-L1 inhibitors reduces osteoclast activity by approximately 60% via inhibition of the JNK signaling pathway, whereas long-term use is associated with an increased risk of fractures (hazard ratio ≈ 1.6). The PD-1/PD-L1–PKM2 axis plays a pivotal role in osteoblast differentiation: PD-L1 enhances osteogenesis by upregulating RUNX2 expression, while PKM2 activation suppresses osteoblast function. The Wnt signaling pathway exerts dual effects—its canonical branch inhibits osteoclastogenesis.

**Figure 2** provides an integrated overview of the bidirectional regulatory mechanisms of the PD-1/PD-L1 axis in bone metabolism and the effects of its inhibition. It illustrates how the PD-1/PD-L1 pathway interacts with multiple signaling axes to regulate both osteoclasts and osteoblasts, and highlights the temporal differences in short- vs. long-term blockade, the influence of the bone microenvironment, and crosstalk with the Wnt/β-Catenin pathway.

## 5 Challenges

### 5.1 Contradictory clinical findings and incomplete mechanistic understanding

Current clinical observations on the impact of PD-1/PD-L1 inhibition on bone metabolism yield conflicting results, and the underlying mechanisms remain poorly elucidated. Differences in clinical characteristics may partly explain these inconsistencies (see **Table 5**). Age and sex are major determinants: elderly female patients treated with ICIs have a significantly increased risk of fractures (21, 31), and baseline bone mineral density (BMD) has been identified as a critical predictor—patients with lower BMD derive less benefit from PD-1 blockade (32). Additionally, treatment regimens (monotherapy vs. combination therapy), tumor type, and tumor burden also influence skeletal outcomes, sometimes resulting in opposing bone metabolic responses (4, 5). These contradictions highlight the complexity of PD-1/PD-L1-mediated regulation of bone metabolism and underscore the urgent need for further research.

**Table 5:** Impact of clinical characteristics on bone metabolism in patients receiving PD-1/PD-L1 inhibitors.

Year	Tumor Type / Cohort (n)	Key Clinical Features Assessed	Bone Metabolic Effects & Core Findings	Mechanistic Implications	Ref.
2020	Breast cancer bone metastasis (mouse model)	Tumor burden (bone metastasis vs. non-metastatic)	PD-1 blockade ↓ osteoclasts by 46%, suppressed bone resorption	PD-1 expressed on osteoclast precursors; blockade → NFATc1 inhibition → anti-resorptive effect	(5)
2023	Various solid tumors (claims database, n = 1,873)	Age (≥65 vs <65), sex, mono- vs. dual-ICI	Major fracture risk ↑ 2.5-fold within 1 year post-ICI; highest risk in elderly females and dual-ICI users	T cell activation → RANKL upregulation → increased bone resorption	(21)
2024	Melanoma (claims database, n = 3,137)	Age (mean 68), sex (36% female), dual ICI, prior fractures	MOF risk HR ≈ 1.8 during years 1–2 post-ICI; higher risk in older age, females, and combination therapy	Systemic immune activation → hyperactive osteoclastogenesis	(31)
2024	Multiple solid tumors (prospective cohort, n = 57)	Mean age 59 ± 13; 57% prior chemotherapy; 21% prior radiotherapy	P1NP ↑ 34%, CTX ↓ 18% at 12 weeks → bone formation favored	PD-1/PD-L1 blockade ↑ CD14^+^ osteoprogenitors, promoting osteogenesis	(4)
2024	NSCLC (n = 229)	Baseline BMD (QCT) and TNM stage III–IV	Low BMD group: PFS HR 1.72, OS HR 1.88 → bone loss correlated with poor ICI outcomes	Bone mass reflects systemic inflammation/nutrition; bone-immune interplay	(32)
2025	Mouse model + patient serum	Age, sex	Young female mice: trabecular bone volume ↓ 33%; males unaffected; aged mice of both sexes showed BMD decline	CD3^+^ T cell–mediated RANKL/OPG imbalance; PD-1 blockade drives T cell–dependent bone loss	(22)
2025	Melanoma (opportunistic QCT, n = 98)	Baseline vBMD, high-dose corticosteroids	vBMD ↓ 6.9 mg/cm³ at 12 months in non-ICI group; stable in ICI group; patients with low baseline vBMD still declined	ICI may preserve bone by suppressing inflammation-driven resorption; corticosteroids counteract benefit	(9)

### 5.2 Lack of clinical monitoring and interventions targeting bone metabolism

Although ICIs are significantly associated with adverse skeletal events—including pathological fractures, vertebral compression fractures, and femoral neck fractures (14)—these complications are often under-recognized in clinical practice. Reports indicate that up to 69% of patients may experience bone-related adverse events, with some requiring extended treatment intervals or premature discontinuation of cancer therapy (15). These are not isolated cases but rather reflect a widespread oversight in clinical monitoring (see **Table 6**). Multiple studies (4, 7) reveal a lack of baseline BMD evaluation and exceedingly low usage of bone-protective agents—fewer than 10% of patients receive treatment for osteoporosis (14, 21). Such gaps in screening and intervention may directly contribute to the occurrence of multiple fractures and bone resorptive lesions (8). Nevertheless, osteoporosis and fragility fractures are not yet formally recognized as immune-related adverse events (irAEs) (16). Baseline and longitudinal assessments of skeletal health are essential and should be systematically implemented in patients undergoing ICI therapy (33).

**Table 6:** Gaps in bone metabolic monitoring during PD-1/PD-L1 inhibitor therapy.

Year	Study Design / Cohort (n)	Baseline BMD Evaluation Rate	Baseline Use of Bone-Modifying Agents	Bone Events or Biomarker Data	Identified Monitoring / Intervention Gaps	Ref.
2018	Case series of 6 “bone irAEs”	0% (no DXA performed before fractures)	0% (ZA or denosumab started post-fracture)	3 new vertebral fractures; 3 focal osteolytic lesions	No baseline BMD assessment or preventive treatment; multiple early skeletal events	(8)
2021	Case series (n = 4) + FAERS pharmacovigilance (n = 650)	0% (no DXA in cases; FAERS lacked data)	<10% reported concurrent use of BMA	FAERS: pathological fracture ROR 3.17; 3/4 case series with multiple vertebral fractures	Large-scale reports lack bone baselines; bone-protective treatment highly underused	(14)
2022	Prospective observational; NSCLC/RCC (n = 44)	0% (DXA/QCT not performed; only CTX-I, P1NP tested)	0% (excluded prior BMA users)	CTX-I ↑, P1NP ↓ within 3 months → increased bone resorption	No imaging-based BMD at baseline; no protective intervention	(7)
2023	Real-world index cohort (Canada; n = 1,600 ICI users)	NR (DXA not recorded in database)	8.8% received anti-osteoporotic drugs before ICI	MOF incidence 27.3/1000 person-years post-ICI; IRR 2.43	>90% without bone protection; no risk stratification applied	(21)
2024	Prospective cohort; ICI monotherapy in various cancers (n = 9)	0% (osteoporotic patients excluded; no DXA)	0% (excluded prior ZA/denosumab use)	CTX ↓ at 1 month; PINP & OCN ↑ at 4–6 months → biphasic metabolic shift	Lack of baseline bone mass assessment limits clinical interpretation of metabolic changes	(4)

## 6 Discussion

Both clinical and mechanistic studies have reported contradictory findings regarding the role of the PD-1/PD-L1 pathway in bone metabolism regulation (4, 6, 10, 18). These discrepancies highlight the complexity of this pathway and the bidirectional effects of its inhibitors on bone homeostasis, suggesting a potential bone-protective role for PD-1/PD-L1 blockade. Compared with traditional bone-protective agents (**Table 7**), PD-1/PD-L1 inhibitors act further upstream in the RANKL axis (5), exerting a dual regulatory effect—initial inhibition of osteoclastogenesis followed by promotion of osteogenesis (4). This complements the unidirectional effects of conventional agents such as bisphosphonates and denosumab (34, 35).

**Table 7:** Distinct effects of PD-1/PD-L1 inhibitors versus traditional bone-protective agents on bone metabolism.

Year	Key Pathway	Drug / Modality	Core Bone Metabolic Effects & Representative Data	Overlaps/ Key Differences	Ref.
2014	IPP–γδ T cell activation axis	Zoledronic acid → γδ T cell	ZA inhibits FPP synthase → IPP accumulation → activates Vγ9Vδ2 T cell degranulation and cytotoxicity against monocyte–myeloid lineage	Unique “immune adjuvant” effect of bisphosphonates; may synergize with ICIs to boost antitumor immunity; primarily anti-resorptive	(37)
2020	RANKL → RANK → NFATc1 (osteoclastogenesis axis)	PD-1 inhibitor (nivolumab)	In murine bone metastasis: anti-PD-1 ↓ osteoclasts by 46%, inhibited bone resorption, relieved pain; blocks PD-1–SHP2 → suppresses NFATc1	Targets same axis as denosumab but upstream (PD-1 vs RANKL); adds immuno-oncologic benefit	(5)
2022	Mevalonate–FPP synthase	Nitrogen-containing bisphosphonates (ZA)	FPP synthase inhibition → IPP/ApppI accumulation → osteoclast apoptosis; CTX ↓ 50–60%, annual BMD ↑ 4–6%	Downstream metabolic action; RANKL-independent; complements both ICI and denosumab	(35)
2023	RANKL → RANK/TRAF6	Denosumab (anti-RANKL mAb)	Phase III trials: delayed skeletal events by 18–23%; CTX ↓ up to 80%	Same ligand target as ICI (RANKL) but acts via direct neutralization; fast, reversible effect; limited immune activation	(34)
2024	T cell activation–derived RANKL	PD-1/PD-L1 antibodies (prospective multicancer cohort)	P1NP ↑ 34% at 12 weeks; 1-year fracture risk ↑ 2.5× (elderly/female at highest risk)	ICI shares ligand (RANKL) with denosumab but shows bidirectional effects; bone protection strategies required	(4)
2024	RANKL blockade + PD-1 blockade combination	Denosumab + ICI	NSCLC with bone metastasis: ORR ↑ from 37% → 54%; skeletal events ↓ 30%; no increase in irAEs	Highlights RANKL–bone and checkpoint–immune synergy; dual benefit in bone protection and tumor control	(36)

Notably, combining PD-1 inhibitors with denosumab has been shown to increase the objective response rate in tumors (from 37% to 54%) and reduce the incidence of skeletal-related events by approximately 30% (36). Similarly, their combination with zoledronic acid can synergistically enhance anti-tumor immunity and anti-osteoporotic effects through the activation of γδ T cells (37). These findings suggest that PD-1/PD-L1 inhibitors may offer a novel therapeutic avenue for osteoporosis prevention and the management of skeletal-related adverse events (SREs) (14, 15).

As evidence continues to accumulate, it is anticipated that within the next five years, osteoporosis and fragility fractures will be increasingly recognized as part of the spectrum of immune-related adverse events (irAEs) associated with immune checkpoint inhibitors (ICIs) (16). Looking further ahead, over the next decade, the integration of bone-targeted strategies with ICI-specific bone-protective protocols may emerge (33), aiming to balance anti-tumor efficacy with bone health preservation and reduce the risk of SREs.

In the coming 5–10 years, an evidence-based framework for comprehensive skeletal health monitoring and stratified prevention is expected to be developed and refined. As summarized in **Table 8**, several emerging findings support this trend: baseline CT-derived BMD values have been shown to correlate with survival outcomes following ICI therapy (32), suggesting that CT-BMD should be incorporated into pre-treatment screening. The newly developed “Mel-ICI Fracture Score” can help identify high-risk individuals, with scores ≥4 warranting the initiation of bone-protective agents (31). Given that fracture risk increases 2.6-fold within the first year of ICI treatment, DXA assessments are recommended at therapy initiation (21). Moreover, AI-assisted QCT techniques, coupled with dual-track monitoring of BMD changes and serum bone turnover markers, can enable precise and timely surveillance of bone health (4, 9).

**Table 8:** Bone Monitoring and Risk-Stratified Prevention Strategies in ICI Therapy.

Year	Design / Population	Key Bone Findings	Implications for Monitoring–Stratification–Prevention	Ref.
2023	Review	Summarized bidirectional bone effects of ICIs; proposed follow-up algorithm	Recommends “Bone-Oncology Joint Clinic”: baseline DXA + BTM → 6-month reassessment → annual review	(38)
2023	Review	ICIs may worsen outcomes in bone metastatic cancer; benefit from bone-targeted co-treatment	Advocates for separate stratification of bone metastasis patients; ICI + anti-resorptive combo and enhanced bone pain surveillance	(39)
2023	Provincial Canadian database; 2,285 ICI-naïve cancer patients	1-year fracture HR 2.6 post-ICI; most involved spine/ribs	Suggests DXA + FRAX at ICI initiation; anti-resorptives for high-risk individuals	(21)
2024	Retrospective–matched cohort; 479 NSCLC patients on ICIs	Low CT-BMD group had shorter OS (~3 months); no impact on PFS	Proposes CT-derived BMD as prescreening tool; integrate low-BMD patients into osteoporosis prevention pathway	(32)
2024	Prospective cohort (n = 30) + 3D bone model validation	After 3 months: CTX ↓ 25%, OCN ↑ 18%	Emphasizes dual-track: serum bone turnover markers + imaging; timely Ca/Vit D supplementation	(4)
2024	Multicenter melanoma retrospective cohort (n = 1,104)	MOF incidence 31.2/1000 person-years (vs. 14.8); shoulder–hip–spine predominant	Proposes “Mel-ICI Fracture Score”; patients scoring ≥4 should initiate bisphosphonates/denosumab	(31)
2025	ML-aided QCT study; 132 ICI patients	Median L1 vBMD ↓ 5.4% at 6 months; AI enabled automated quantification	Embeds AI-QCT into routine imaging to enable longitudinal monitoring and personalized alerts	(9)

**General Table Notes:** Symbols used across all tables are defined as follows: ↑ indicates increase or upregulation; ↓ indicates decrease or downregulation; --/-- denotes gene knockout; ± indicates the presence or absence of a treatment or condition; → means “leads to” or “results in”; ≈ indicates approximate value; + indicates marker positivity; and × denotes **fold change relative to baseline or control**. All numerical values represent changes from control or baseline conditions and are expressed as percentage, fold change (×), or absolute values, as specified in each table. **Abbreviations used throughout include:** BMD (bone mineral density), CTX(-I) (C-terminal telopeptide of type I collagen), P1NP (N-terminal propeptide of type I procollagen), TRAP (tartrate-resistant acid phosphatase), ALP (alkaline phosphatase), OCN (osteocalcin), RUNX2 (Runt-related transcription factor 2), COL1A1 (collagen type I alpha 1), BV/TV (bone volume to total volume ratio), MAR/BFR (mineral apposition rate/bone formation rate), DXA (dual-energy X-ray absorptiometry), QCT (quantitative computed tomography), HR (hazard ratio), CI (confidence interval), IPP/FPP/ApppI (isoprenoid pathway intermediates), PKM2 (pyruvate kinase M2), OxPhos (oxidative phosphorylation), OE/KD (overexpression/knockdown), mAb (monoclonal antibody), BMA (bone-modifying agents), MOF (major osteoporotic fracture), FAERS (FDA Adverse Event Reporting System), FRAX (Fracture Risk Assessment Tool), CT-BMD (CT-derived BMD), AI (artificial intelligence), and ML (machine learning).

The establishment of a “Bone-Oncology Joint Clinic” model is also proposed, facilitating a closed-loop management approach—from baseline evaluation to regular follow-up and comprehensive intervention (38), as well as stratified care for patients with bone metastases (39). These innovations aim to address bone loss and provide continuity of care for patients who develop skeletal complications during ICI treatment (15), ultimately mitigating poor prognostic outcomes (7). Collectively, these strategies may enable dual optimization of tumor control and skeletal health during ICI therapy and significantly improve the quality of life and long-term health outcomes of cancer survivors.

whereas the non-canonical branch promotes osteoclast formation. Clinical observations suggest that immune checkpoint inhibitors primarily promote bone formation indirectly by suppressing osteoclast activity rather than by directly enhancing osteoblast differentiation.

## Data Availability

The original contributions presented in the study are included in the article/supplementary material. Further inquiries can be directed to the corresponding author.
